# Effect of *Elaeagnus umbellata* (Thunb.) fruit extract on H_2_O_2_-induced oxidative and inflammatory responses in normal fibroblast cells

**DOI:** 10.7717/peerj.10760

**Published:** 2021-01-19

**Authors:** Klara Zglińska, Tomasz Niemiec, Andrzej Łozicki, Magdalena Matusiewicz, Jarosław Szczepaniak, Kamila Puppel, Marta Kutwin, Slawomir Jaworski, Anna Rygało-Galewska, Piotr Koczoń

**Affiliations:** 1 Institute of Animal Science, Warsaw University of Life Sciences, Warsaw, Poland; 2 Institute of Biology, Warsaw Univeristy of Life Sciences, Warsaw, Poland; 3 Institute of Food Science, Warsaw University of Life Sciences, Warsaw, Poland

**Keywords:** *Elaeagnus umbellata*, Autumn olive, Plant extract, Anti-oxidative, Anti-inflammatory, Fibroblast, Hydrogen peroxide

## Abstract

**Background:**

*Elaeagnus umbellata* is a plant commonly used in traditional Asian medicine for its many health benefits and strong antioxidative activity. Its therapeutic potential is believed to be connected to its effect on fibroblasts. This study aimed to investigate *E. umbellata* methanol-acetone extract’s (EUE) defense against hydrogen peroxide (H_2_O_2_)-induced fibroblast damage.

**Methods:**

Because the main biologically active compounds of *E. umbellata* are water-insoluble, we evaluated the effects of methanol-acetone fruit extracts using liquid chromatography (for ascorbic acid and beta-carotene) and spectrophotometry (for lycopene and total phenolics). The extract’s antioxidative activity was measured using DPPH radical inhibition, and EUE’s effect on human fibroblasts was also evaluated. We assessed the metabolic activity and apoptosis of HFFF-2 fibroblasts exposed to EUE and/or H_2_O_2_using the XTT test and flow cytometry, respectively. Superoxide dismutase activity and reactive oxygen species (ROS) production were evaluated using colorimetric and fluorometric assays, respectively. We measured pro-inflammatory cytokine (MIF, fractalkine, MCP-4, BLC, GCP-2, NAP-2, Eotaxin-2, and Eotaxin-3) expression in HFFF-2 cells using immunocytochemistry.

**Result:**

The extract increased HFFF-2 cell proliferation and reduced cell death caused by H_2_O_2_-induced stress. H_2_O_2_-treated fibroblasts had greater ROS production than cells treated with both H_2_O_2_ and EUE. Additionally, the group treated with H_2_O_2_ alone showed higher pro-inflammatory cytokine (MIF, MCP-4, NAP-2, Eotaxin-2, and Eotaxin-3) expression.

**Conclusion:**

EUE protected human fibroblasts from H_2_O_2_-induced oxidative stress and reduced the fibroblast-mediated inflammatory response triggered by ROS.

## Introduction

Fibroblasts are the most common type of cells in mammalian connective tissue. In addition to their role as a structural ingredient, fibroblasts contribute to various immune and inflammatory responses. Human chronic inflammatory diseases can develop into a number of debilitating fibrotic disorders, proving that permanent activation of the immune system can lead to strong disturbance in fibroblast function (Williams, 1998; Mescher, 2017). Fibroblasts respond to the actions (attendance) of many types of cytokines, and these mediators trigger fibroblasts and leucocytes to act together during complex healing processes such as wound healing. Fibroblasts can modify the chemical composition of their secretions in response to other cell mediators (Mescher, 2017). Additionally, fibroblasts are frequently exposed to inflammatory agents related to infections and UV or ionizing external radiation exposure. It is well known that any type of physical injury in multicellular animals immediately produces a cascade of enzymatic and cellular reactions within the destructed tissues (Watanabe et al., 2004). Therefore, compounds that can protect fibroblasts against oxidative stress and inflammation are extremely important.

The *Elaeagnus* genus includes 70 to 80 species, the most studied of which being *Elaeagnus angustifolia* L. (Russian olive). Another oft-mentioned variety is *Elaeagnus umbellata,* frequently referred to as the autumn olive, silverberry, or spreading oleaster. It can be found in Asia, North America, or its place of origin, Southeast Asia. The *Elaeagnus sp*. berry is an excellent source of antioxidative compounds, including carotenoids, phenolic, flavonoids, and vitamins, specifically vitamins A, C, E, and D (Uddin & Rauf, 2012; Khattak, 2012; Hamidpour et al., 2016; Ozen et al., 2017). The carotenoids beta-carotene and lycopene have proven antioxidative and anti-cancer effects. The carotenoids and *α*-tocopherol work with reactive oxygen species (ROS), especially peroxyl radicals, and singlet oxygen. Beta-carotene and lycopene act as antioxidants during lipid phases by neutralizing singlet oxygen or ROS (Sies & Stahl, 1995). Vitamin E mediates cell signaling and proliferation, as well as gene expression regulation. *α*-tocopherol, the most prevalent form of this vitamin in human tissue, exhibits antioxidant and anti-inflammatory properties (Patel, 2015).

Lycopene is the main compound in tomatoes, and is found in six to 16 times greater quantities in autumn olive berries (Fordham et al., 2001; Patel, 2015). *Elaeagnus* fruits are also a good source of high-quality oils. Fat extracted from *E. augustiflora* seeds mostly consists of unsaturated fatty acids, including linolenic acid, with well-tested strong anti-inflammatory properties (Hamidpour et al., 2016).

Due to the complex nature of *E. umbellata*’s antioxidative mechanism, a combination of methods should be applied in order to correctly determine its complete properties. The reduction of ROS secretion by cells subjected to oxidative stress also confirms the effects of these antioxidant properties. Under certain physiological conditions, ROS are present in the cell at very low levels and only execute signalling functions. The increase of ROS intracellular levels results from oxidative stress (Schieber & Chandel, 2014). ROS secretions can be measured in vitro in living cells using a test in which ROS react with a fluorogenic sensor localized in the cytoplasm, resulting in a fluorometric product amount that is proportional to the amount of ROS present in the studied sample (Cusimano et al., 2017).

Hydrogen peroxide (H_2_O_2_) is mainly generated from superoxide anions produced by mitochondria and NADPH oxidases. Physiological levels are involved in normal cell metabolism, whereas increased levels of superoxide or hydroxyl radicals can invoke oxidative stress. Accordingly, H_2_O_2_ has been shown to be involved in the formation of numerous pathologies. More specifically, H_2_O_2_ can exert its toxic effects through the ferrous iron-dependent formation of highly reactive hydroxyl radicals, which in turn can alter the structure of lipids, proteins, and DNA (Halliwell & Gutteridge, 1992). Oxidative stress can trigger the activation of cytokines or chemokines, as well as the aggregation of metabolites resulting from oxidation. Many oxidative stress promoters recruit inflammatory cells either directly or indirectly, therefore increasing ROS production and creating an increased inflammatory state. Oxidative stress may be critical during the activation of nuclear factor kappa-light-chain-enhancer of activated B cells (NF- κB), based on the observations that the cytokine-induced activation of NF- κB could be prevented by antioxidant or metal chelator treatment (Nagata, 2005). The effects of H_2_O_2_ on epidermis cells was found to vary depending on the dose amount. Song et al. (2012) showed that concentrations of H_2_O_2_ over 100 µM significantly increased the expression of tumour necrosis factor *α* (TNF-*α*) mRNA in human middle ear epithelial cells, suggesting that this dose was appropriate for studying anti-inflammatory effects.

Inflammation, controlled by chemotactic cytokines known as chemokines, manifests itself in many diseases such as arthritis, meningitis, respiratory system disorders, and neurodegeneration. These disease’s chemokines can trigger leukocyte accumulation and activation in tissues. By controlling the movement of inflammatory cells, previous studies have proposed that different chemokines and their receptors could provide direction for future therapeutic interventions (Luster, 1998; Nagata, 2005).

Chemokines have been found to play an important role in connective tissue diseases by recruiting eosinophils to the inflammation site and releasing ROS, causing tissue damage and propagating the inflammatory response. One therapeutic strategy to reduce excessive inflammatory responses is to inhibit chemokine activity or block their receptors (Waśniowska, 2004). Both inflammation and connective tissue injuries have also been associated with collagen degradation, a process where matrix metalloproteinase proteins (MMPs) are activated. Endothelial cells and monocytes secrete MMPs in response to various inflammatory stimuli and oxidative stress (Galis et al., 1998; Calabriso et al., 2016).

Additionally, there is evidence of the involvement of MMP-2 and MPP-9 during neurodegeneration. Under particular neuro-inflammatory conditions, MMPs have been shown to change the permeability of the blood-brain barrier, resulting in white matter damage. Therefore, MMPs may be related to amyloid formation in Alzheimer’s disease, as well as the death of dopaminergic neurons in Parkinson’s disease (Rosenberg, 2009). The number of neurons in the brain has been found to increase in the presence of hepatocyte growth factor (HGF), which has also been shown to regulate the migration and mobility of newly-created cells (Respondek & Buszman, 2015).

When searching for natural products that are able to moderate oxidative stress and inflammation, traditional medicine provides several options. The *E. umbellata* berry has been used in traditional Asian medicine as an anti-inflammatory and anti-nociceptive agent (Uddin & Rauf, 2012; Ahmad et al., 2009; Hamidpour et al., 2016; Ozen et al., 2017). Its antidiabetic (Nazir et al., 2018; Spínola et al., 2019) and anti-proliferative (Wang, Bowman & Ding, 2007; Ozen et al., 2017) potential have been also demonstrated. However, scientific data on the molecular mechanisms of *Elaeagnus sp.* extracts’ antioxidative and anti-inflammatory effects remain limited. To our knowledge, there have been no studies on the effects of autumn olive fruit extract on normal human fibroblast cells.

In this study, we investigated the potential antioxidative and anti-inflammatory effects of *E. umbellata* fruit extract during ROS production and inflammation-associated cytokine expression in H_2_O_2_-induced human fibroblast cells.

## Materials & Methods

### Samples

Fruits of *Elaeagnus umbellata* were collected from a farm located in the Łódź Voivodeship in central Poland. Fruits were harvested after ripening. The species classification was confirmed by Piotr Banaszczak (Rogów Arboretum, Warsaw University of Life Sciences, Poland). The fruits were then lyophilized.

A field permit was not required as the fruit was bought at an ornamental plant farm.

### Extract preparation

First, samples were incubated with methanol and acetone (1:50:10) in an ultrasound bath for 30 min. Then, extracts were filtered and the process of extraction was repeated. Extracts were combined and methanol was evaporated under vacuum in a rotary evaporator (40 °C). Next, they were kept in −20  °C until analysis. All extraction procedures were performed in darkness.

### Extract characteristics

#### High-performance liquid chromatography

Fat extraction and saponification of *E. umbellata* berry extracts was performed according to the AOAC procedure (1990) at 25  °C. Analysis of carotenoids, beta-carotene, D (group), E (*α*-tocopherol), K (phylloquinone) in crude fat was conducted using an Agilent 1,100 high performance liquid chromatography (HPLC) system (Agilent, Waldbronn, Germany). Separations were performed at 25  °C using a 4.6 × 150 mm and 5 µm Zorbax Eclipse XDB C8 column (Agilent). Solvent flow rate was maintained at 1.2 mL/min in a linear gradient of 90:10 (vol./vol.) methanol to water, with an injection volume of 10 µL. The temperature of the column oven was set at 30  °C. Detection with a UV–vis detector was carried out at 230 nm for *α*-tocopherol, and 265 nm for K, carotenoids, β-carotene, and vitamin D. The standard solution (1 mg/mL) was prepared separately by dissolving 10 mg of each individual vitamin in 10 mL of methanol and storing in darkness at 4  °C. *N* = 3

L-ascorbic acid levels was determined by HPLC using the method described by Odriozola-Serrano, Hernández-Jover & Martín-Belloso (2007). Briefly, 0.1 g of the extract was transferred into a volumetric flask and filled up to 10 mL of pure ethanol. A part of the mixture was filtered through a 0.45 µm PTFE syringe filter onto a chromatographic vial and 20 µL were analyzed using HPLC, an Onyx Monolithic C18 column (100 × 4.6 mm, 5 µm; Phenomenex, Torrance, CA, USA), and a UV-Vis detector (Shimadzu, Kyoto, Japan). The mobile phase was 0.1% m-phosphoric acid solution in ultrapure water. The analysis was carried out using isocratic conditions ( one mL/min) at a 25  °C column temperature, and detection was performed at 254 nm. The calibration curve was performed with L-ascorbic acid external standard solutions (*N* = 3).

#### Phenolic content

The total phenolic (TC) content was determined using the standard Folin–Ciocalteu method. The extract was was combined with 2% sodium carbonate in deionized water and vortexed the mixture well. Next, 50% Folin–Ciocalteau reagent was added to the mixture and vortexed. It was left to settle for 45 min and the absorbance was measured at 750 nm. TC contents were calculated with the use of calibration curve of quercetin. The results were presented as quercetin equivalent milligram per gram (mg/g) of *E. umbellata* extract dry matter (d.m.; *N* = 6).

#### Lycopene content

The spectrophotometric method was used to determine the amount of lycopene (Rao, Waseem & Agarwal, 1998). Lycopene was extracted from each sample using a mixture of butylated hydroxytoluene (BHT) in acetone, ethanol and hexane (1:1:2) and values were determined by measuring absorbance at λ = 503 nm in the hexane layer. The lycopene content was calculated using the lycopene extinction coefficient in hexane, at this wavelength (*N* = 3).

### Antioxidant potential

#### *α*, *α*-diphenyl- β-picrylhydrazyl (DPPH) free radical scavenging

The anti-oxidant in vitro properties of extracts were measured using DPPH free radical scavenging test, as previously shown by Matusiewicz et al. (2009). Concentrations ranging from 100 to 1,000 µg/mL were used and the results were expressed as a percentage of DPPH inhibition. Total extract and *α*-tocopherol were diluted in methanol and L-ascorbic acid in deionized water before the analysis.

Regarding the scavenging activity of the DPPH? test, 0.2 mM DPPH? in methyl alcohol was mix with each extracts or standard antioxidant (L-ascorbic acid and *α*-tocopherol). After 30 min in darkness (25 °C), samples were centrifuged (1,600 g, 10 min) and then the absorbance was measured at 517 nm (*N* = 6).

### Cell cultures and treatments

The HFFF-2 human foetal foreskin fibroblast cell line was obtained from the European Collection of Authenticated Cell Cultures (ECACC, 12 passage) and maintained in Dulbecco’s modified medium (DMEM, Gibco, Thermo Scientific, Waltham, MA, USA) supplemented with 10% foetal bovine serum (Sigma-Aldrich/Merck, Darmstadt, Germany), 1% penicillin, and streptomycin (Sigma-Aldrich/Merck, Darmstadt, Germany) at 37  °C in a humidified atmosphere of 5% CO_2_/95% air in a NuAire DH AutoFlow CO_2_ Air-Jacketed Incubator (Plymouth, MN, USA). Cells that reached about 70% confluence were used for the experiment.

### The effect of extracts on cell viability

To measure cell viability, the XTT Kit (Sigma-Aldrich/Merck, Darmstadt, Germany), a colorimetric assay for the nonradioactive quantification of cellular proliferation, viability, and cytotoxicity, was used. The assay is based on the extracellular reduction of XTT by NADH produced in the mitochondria. XTT is only reduced to formazan by metabolically active cells. Cells were plated into 96-well plates at 17 000 cells/well and 100 µL DMEM was added to each well. After 24 h, 90 µL of fresh culture medium and 10 µL of extract at concentrations ranging from 100 to 500 µL/mL were added to each well.

The following groups were created:

I. Control without extract or H_2_O_2_

II. 100 µg/mL of extract without H_2_O_2_

III. 150 µg/mL of extract without H_2_O_2_

IV. 200 µg/mL of extract without H_2_O_2_

V. 250 µg/mL of extract without H_2_O_2_

VI. 500 µg/mL of extract without H_2_O_2_

VII. 100 µg/mL of extract with H_2_O_2_

VIII. 150 µg/mL of extract with H_2_O_2_

IX. 200 µg/mL of extract with H_2_O _2_

X. 250 µg/mL of extract with H_2_O_2_

XI. 500 µg/mL of extract with H_2_O_2_

XII. H_2_O_2_ alone.

The extract was mixed with PBS and stirred until completely dissolution. Equal volumes of PBS were added to control cells. After 72 h, 100 µM H_2_O_2_ was added to induce oxidative stress.

Then, after another 24 h, the XTT test was performed according to the manufacturer’s protocol.

Optical density (OD) was measured at 450 nm using an enzyme-linked immunosorbent assay reader (Infinite M200, Tecan, NC, USA). Cell viability was expressed as the percentage of (ODtest −ODblank)/(ODcontrol −ODblank), where “ODtest” is the OD of cells incubated with the extract, “ODcontrol” is the OD of the control sample, and “ODblank” is the OD of the wells without cells (*N* = 6).

### The effect of the extract and H_2_O_2_ on cell death type

Annexin V/PI Apoptosis Kit (Thermo Fisher Scientific, Waltham, MA, USA) was used to estimate cell death pathways according to Szczepaniak et al. (2018). HFFF-2 cells were seeded in six-well plates at a density of 2 × 10^5^ cells/well. After 24 h, the culture medium was changed to one containing 1% fetal bovine serum (FBS) and 100 µg/mL of 10% extract in PBS (two groups, six wells) or PBS alone (two groups, six wells). After 72 h, 100 µM of H_2_O_2_ was added to two groups (one with extract and another without extract). After 6 h of incubation, the cells were collected and centrifugated. Each cell pellet was then mixed with 100 µL of Annexin binding buffer. 5 µL of fluorescein isothiocyanate-labelled Annexin V and 1 µL of propidium iodide were pipetted into each vial. The samples were left for 15 min at 25  °C. Finally 400 µL of binding buffer and the samples on ice. A total of 10,000 events were recorded per sample and the apoptosis plots were generated using Flowing Software 2.5.1 (Turku Bioscience, Turku, Finland).

This protocol was repeated twice and in triplicate with H_2_O_2_ incubation times of 12 and 24 h, respectively.

### ROS generation

To measure cell-generated ROS, the fluorometric intracellular ROS assay kit (MAK143; Sigma, St Louis, MO, USA)was used. The following groups were created:

I. Control without extract or H_2_O_2_

II. 100 µg/mL of extract with H_2_O_2_

III. 500 µg/mL of extract with H_2_O_2_

IV. H_2_O_2_ alone.

HFFF-2 cells were seeded at a density of 1,700 cells/mL on a 96-well plate with 100 µL of DMEM added to each well. The next day, the medium was removed and replaced with fresh medium containing 1% FBS and dilutions of *E. umbellata* extract at concentrations of 100 mg/L and 500 mg/L in PBS or appropriate volume of PBS. After 72 h, oxidative stress was induced by addition of 100 µM H_2_O_2_ to the groups receiving the extract and the group treated only with H_2_O_2_. H_2_O_2_ dose was chosen based on literature data (Szczepaniak et al., 2018). The controls were treated with PBS. Master Reaction Mix was added into the cell plates and fluorescence intensity was measured at λex520 nm/λem605 nm with a microplate reader.

ROS production was measured every 10 min for a total of six times.

### Superoxide dismutase activity (SOD)

For the SOD activity assay, the following groups were created:

I. Control without extract or H_2_O_2_

II. 100 µg/mL of extract with H_2_O_2_ III. H_2_O_2_ alone.

Cells were seeded at 75 cm^3^ flask in complete medium with the FBS reduced to 1%. After 24 h, extract (100 µg/mL in PBS) or an equivalent volume of PBS was added to cells. After 72 h incubation, oxidative stress was induced by addition of 100 µM H_2_O_2_. Cell pellets from individual groups were pooled to obtain three samples: 100 µg/mL extract + H_2_O_2_, H_2_O_2_ alone, and control. Total protein was isolated and measured using the BCA method. The same amount of protein was used from each group. The isolated protein was analyzed using the SOD determination KIT (Sigma-Aldrich) according to the manufacturer’s instructions. This KIT allowed us to evaluate SOD activity by utilizing WST-1 [2-(4-Iodophenyl)- 3-(4-nitrophenyl)-5-(2,4-disulfophenyl)- 2Htetrazolium, monosodium salt], a highly water-soluble tetrazolium salt that produces a water-soluble formazan dye when reduced with a superoxide anion. The O_2_ reduction rate was linearly related to xanthine oxidase (XO) activity and was inhibited by SOD. The results were expressed as SOD activity (%), according to protocol.

### Cytokine antibody microarrays

The expression of selected cytokines was measured using cytokine arrays, which are antibody-pair-based assays that are analogous to Western blots, but using a membrane as a substrate.

The following groups were created:

I. 100 µg/mL of extract with H_2_O_2_

II. H_2_O_2_ alone

III. Control –PBS only.

Cells at 75 cm^3^ flask in complete medium as described in section ‘Cell cultures and treatments’, except for the FBS concentration, which was reduced to 1%. After 24 h, extract (100 µg/mL in PBS) or an equivalent volume of PBS was added to cells. Following a 72 h incubation period, oxidative stress was induced by adding 100 µM H_2_O_2_. Cell pellets from individual groups were pooled to obtain 3 samples: 100 µg/mL extract + H_2_O_2_, H_2_O_2_ alone, and the control group. Total protein was isolated and measured using the BCA method. The isolated protein was analyzed using the human cytokine antibody array (Abcam), according to the manufacturer’s instructions. he same amount of protein was applied to each membrane. Chemiluminescence detection was performed using the ChemiDoc1 Imaging System and Quantity One Basic Software (Bio-Rad, Hercules, CA, USA). The captured images of each membrane were analysed using Fiji software (National Institute of Health, Bethesda, MD, USA). The results from each group were optimized according to the Abcam protocol (*N* = 4).

### Statistical methodology

Statistical analyses were performed using the Statistica 13 program.

In order to determine antioxidant potential and viability statistical significance of differences between control and individual concentrations of the extract was examined using the ANOVA test. Equality of variances was confirmed using the Brown-Forsythe test. The differences between individual concentrations were checked using the Tukey’s post hoc test.

## Results

### Extract bioactive compounds

*E. umbellata* fruit is known to contain large amounts of phenolic compounds, carotenes, ascorbic acid, and fat-soluble vitamins. ethanol-acetone extracts from *E. umbellata* fruit were evaluated regarding total phenols [1749 (± 5.2) mg], lycopene [50 (± 0.2) mg], beta-carotene [73 (± 0.2) mg], and vitamins D [1.38 (± 0.1) mg], E (*α*-tocopherol) [1124 (± 3.2) mg], K [0.44 (± 0.08) mg], and C (L-ascorbic acid) [64 (± 1.8) mg] levels per 100 g of d.m.

### *E. umbellata* extract antioxidant potential

#### *α*, *α*-diphenyl- β-picrylhydrazyl free radical scavenging

**Figure 1:**
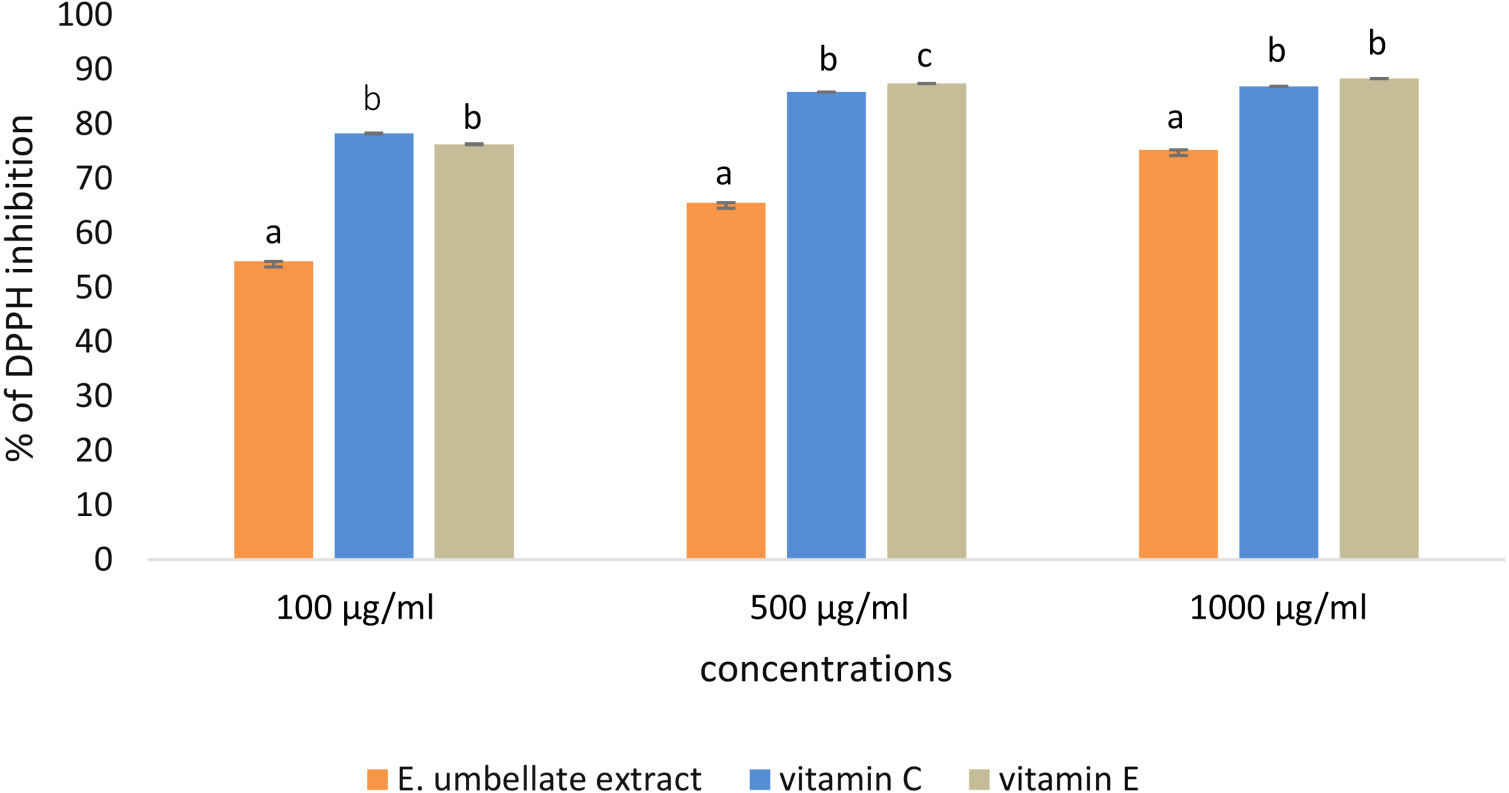
Antioxidant potential of *E. umbellata* extract and standard antioxidants in 100 µg/ml, 500 µg/ml and 1,000 ug/ml concentration. DPPH inhibition is expressed on the y axis in %. Different letters mean that the values for the bars differ statistically in the same concentration (*p* < 0.05) *N* = 6.

DPPH scavenging activity was dose-dependent in the tested concentrations (100–1,000 µg/mL) of *E. umbellata* fruit extract and the standard antioxidants. The results were expressed as the % of DPPH inhibition (Fig. 1).

L-ascorbic acid and *α*-tocopherol had similar antioxidant activity. The extract showed lower potential to reduce DPPH when compared to the standard antioxidants; the extract showed 54.78% potential in the 100 µg/ml dose versus 78.30% for vitamin C and 76.31% for vitamin E. However, this difference was less significant in a higher dose (1,000 µg/ml), reaching 75.25% potential for the extract and 86.24% and 88.37% for L-ascorbic acid and *α*-tocopherol, respectively.

### Influence of extract and H_2_O_2_ on HFFF-2 cell viability

**Figure 2:**
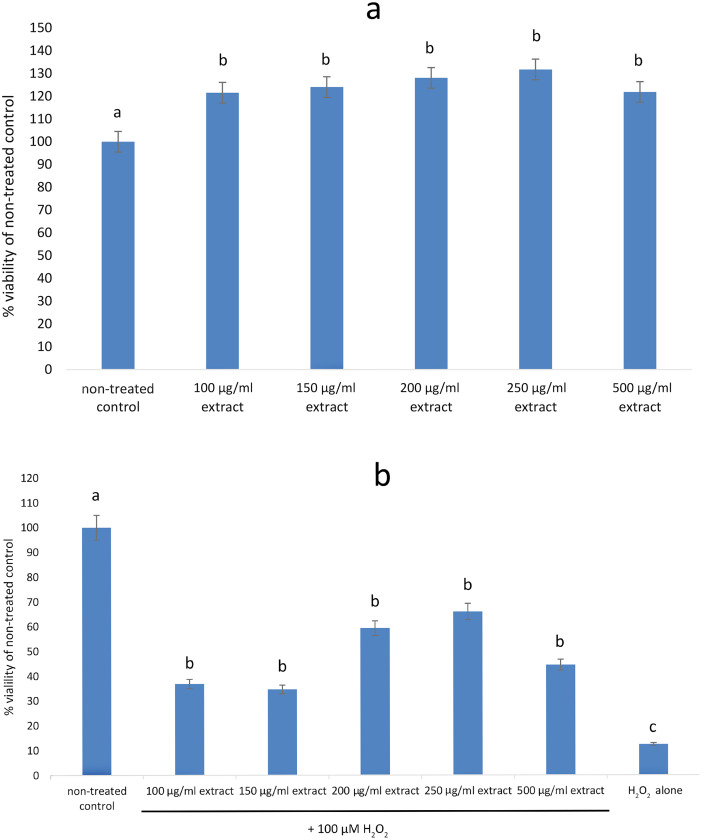
Viability of HFFF-2 cell treated with *E. umbellata* extract and/or H_2_O_2_. (A) Cell treated by various concentration of extract for 72 h. (B) Cell treated by 100µM H_2_O_2_ after 48 h incubation with extract. Non-treated control and H_2_O_2_ only were incubated with PBS instead of extract. Results are expressed as % of non-treated control viability. Mean ± SD. Different letters mean that the values for the bars differ statistically (*p* < 0.05) *N* = 6.

None of the tested concentrations (100–500 µg/mL) of the *E. umbellata* fruit extract showed any cytotoxic effects on HFFF-2 fibroblast cells. After 72 h of treatment, all of the extract concentrations increased cell viability when compared to the non-treated control. The observed effect was statistically significant (*p* < 0.05) across all tested doses (Fig. 2A). In contrast, addition of H_2_O_2_ decreased cell viability by 87.64% as compared to control. Pre incubation with extract for 48 h significantly protect cells from cell death. This effect was statistically significant across all tested doses (Fig. 2B). The differences in effects shown between the 100 µg/mL and higher doses were not statistically significant. Based on this result, we considered the 100 µg/mL dilution the most suitable for testing.

### *E. umbellata* extract rescues fibroblasts from H_2_O_2_-induced cell death

Table 1 shows the ratio of necrosis to apoptosis across all dead cells. Apoptosis was predominant, but the data clearly show that the cells receiving only H_2_O_2_ had the highest rate of necrosis in the total mortality. Across different time intervals, the control or extract-only cells had the lowest necrosis rates, and the highest proportion of necrosis was found in the H_2_O_2_-only cells.

**Table 1:** Determining the type of death of HFFF-2 cells treated with the extract and / or hydrogen peroxide.

Type of cell death after different time of H_2_O_2_ treatment (%)	Control	Extract only	H_2_O_2_ only	Extract + H_2_O_2_	*p*-value
6 h					
Necrosis	0.60^a^	0.77^b^	5.20^c^	2.46^b^	0,000234
Apoptosis	3.38^a^	2.80^b^	7.04^c^	6.65^b^	
Live	96.07^a^	96.62^b^	87.55^c^	91.34^b^	
12 h					
Necrosis	1.02^a^	0.57^a^	17.62^b^	6.56^c^	0,000292
Apoptosis	3.77^a^	2.54^a^	21.67^b^	13.17^c^	
Live	51.11^a^	97.4^a^	60.74^b^	80.26^c^	
24 h					
Necrosis	0.38^a^	0.47^a^	38.47^b^	11.64^a^	0,000231
Apoptosis	2.05^a^	2.12^a^	62.06^b^	43.55^a^	
Live	97.57^a^	97.42^a^	17.05^b^	44.81^a^	

**Notes.**

Groups: control - untreated, extract only - treated with 100 µg / ml extract without H_2_O_2_, H_2_O_2_ only - treated with 100 µM hydrogen peroxide without the addition of extract, extract + H_2_O_2_ - treated with 100 µg/ml extract and 100 µM hydrogen peroxide. Mean ± SD followed by different letters (a,b,c) in the same verse were significantly different for *p* < 0, 05 (post hoc test).

### The extract’s influence on ROS production in HFFF-2 cells

**Figure 3:**
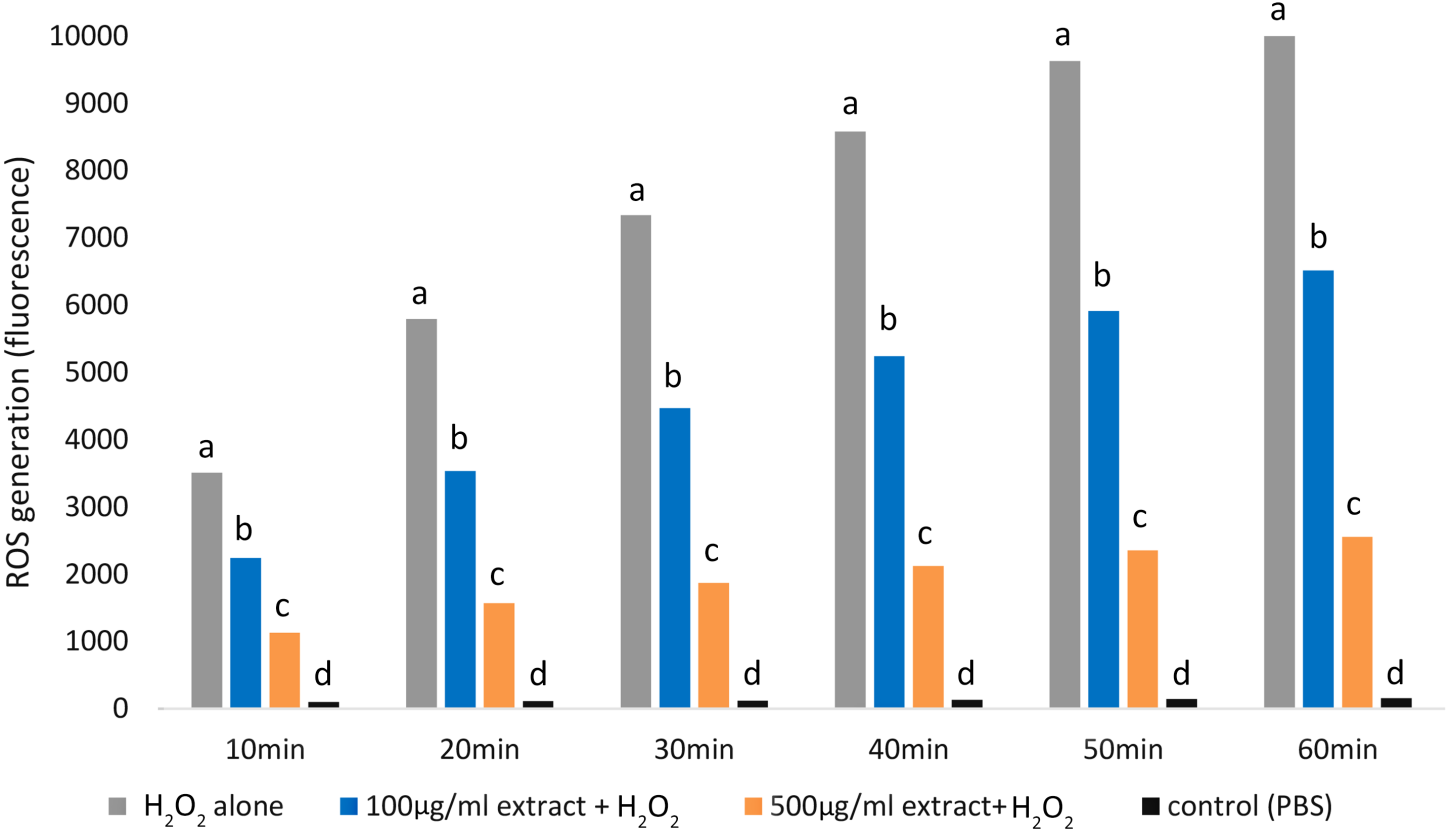
Variations in ROS generation by fibroblasts relative to incubation time and concentrations of the extract of *E. umbellata* fruits. Groups: 500 mg/L extract + H_2_O_2_, 100 mg/L extract + H_2_O_2_, and H_2_O_2_ alone were induced with 100 µM H_2_O_2_ after 72 h of pre-treatment with extract or PBS (H_2_O_2_ alone group). A negative control (not H_2_O_2_-induced) was also included. Different letters mean that the values for the bars differ statistically in the same time interval (*p* < 0.05) *N* = 6.

Adding both *E. umbellata* extract concentrations to cells 72 h before oxidative stress induction resulted in a statistically significant (*p* < 0.05) decrease in ROS production (Fig. 3).

Notably, the 500 mg/L extract concentration had a greater effect in inhibiting ROS production than the 100 mg/L concentration (*p* < 0.05).

### *E. umbellata* extract reduced SOD H_2_O_2_-induced activity.

**Figure 4:**
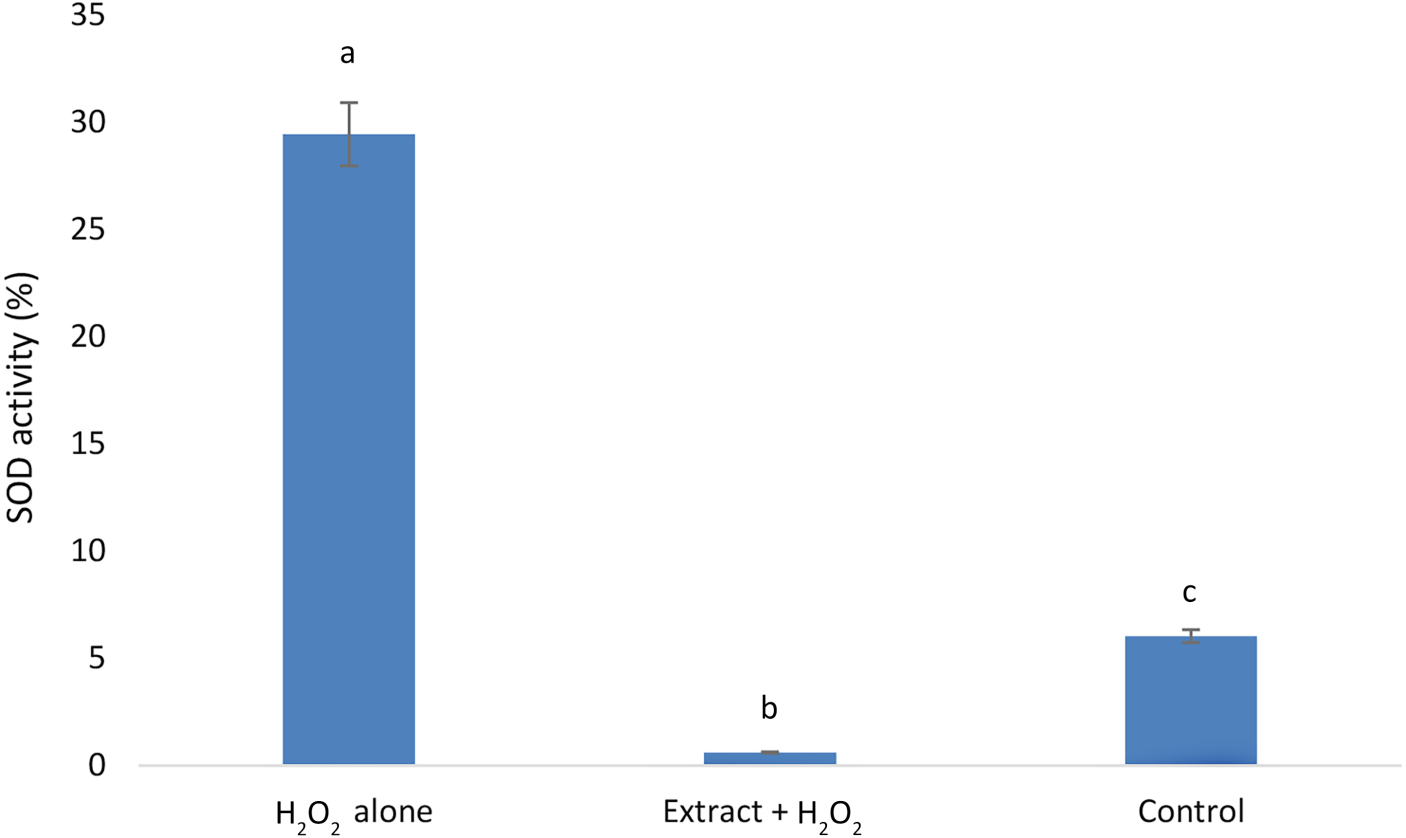
Superoxide dismutase activity in HFFF-2 fibroblast cells treated with *E. umbellata* extract and/or H_2_O_2_. Groups: H_2_O_2_ alone –cells treated only with 100 µM H_2_O_2_. Extract + H_2_O_2_ –cells treated with 100 µg/mL extract of E. umbellata (day 2) and 100 µM H_2_O_2_ (day 5). Non-treat control–control without extract and/or H_2_O_2_. Values expressed as mean ± SD. Different letters mean that the values for the bars differ statistically (*p* < 0.05) *N* = 6.

Our results showed that the H_2_O_2_alone group exhibited the highest SOD activity, followed by the control group. The lowest SOD activity was observed in the group that received *E. umbellata* extract 72 h before the addition of H_2_O_2_ (Fig. 4).

### The *E. umbellata* extract changes the expression of selected growth factors in H_2_O_2_-induced HFFF-2 cells

The use of the extract 72 h before the induction of oxidative stress resulted in changes in chemokine expression in the cells that did not receive the extract and the non-treated control group (Table 2).

**Table 2:** Expression of selected chemokines in HFFF-2 cells after 72 h treatment with 100 µg/ml extract of *E. umbellata* fruit. Oxidative stress was induced by adding 100 µM H_2_O_2_ 24 h before cell lysis.

Group	Extract + H_2_O_2_	H_2_O_2_ alone	Control	*p*-value
BDNF	308.63 ±11.1^a^	135.83 ±1.1^b^	0.00 ±0.0^c^	0.000000
FGF-7	63.64 ±0.4^a^	31.97 ±2.8^b^	32.75 ±5.7^b^	0.000001
HGF	157.09 ±1.6^a^	150.30 ±0.6^b^	45.60 ±2.8^c^	0.001910
TIMP-1	1898.01 ±39.8^a^	1371.93 ±61.2^b^	711.88 ±37.9^c^	0.000000

**Notes.**

H_2_O_2_ alone–cells not incubate with extract; control–cell treated only with PBS (no extract, no H_2_O_2_). *N* = 4; *p*-value–analysis of variance (ANOVA). Mean ± SD followed by different letters (a,b,c) in the same verse were significantly different for *p* < 0, 05 (post hoc test).

More specifically, H_2_O_2_-induced HFFF-2 cells treated with the extract showed lower expressions of macrophage migration inhibitory factor (MIF), fractalkine, monocyte chemoattractant protein 4 (MCP-4), B lymphocyte chemoattractant (BLC), granulocyte chemotactic protein 2 (GCP-2), neutrophil-activating peptide 2 (NAP-2), Eotaxin-2, and Eotaxin-3 than in cells treated only with H_2_O_2_. The non-treated control samples showed the lowest expression of chemokines, of which fractillin, BLC, GCP-2, and Eotaxin-3 were negligible. Cells treated with the extract and H_2_O_2_ had more than twice the expression of fibroblast growth factor 7 (FGF-7) and brain-derived neurotrophic factor (BDNF) after H_2_O_2_ administration than cells treated only with H_2_O_2_. Likewise, TIMP metallopeptidase inhibitor 1 (TIMP-1) and HGF expression was higher by over 40% and 5%, respectively (Table 3). The non-treated control samples had the lowest expression of these factors, and cells that were not exposed to the extract had undetectable BDNF levels (Table 3).

**Table 3:** Expression of selected cytokines in HFFF-2 cells after 72 h treatment with 100 µg/ml extract of *E. umbellata* fruit. Oxidative stress was induced by adding 100 µM H_2_O_2_ 24 h before cell lysis.

Group	Extract + H_2_O_2_	H_2_O_2_ alone	Control	*p*-value
BDNF	308.63 ±11.1^a^	135.83 ±1.1^b^	0.00 ±0.0^c^	0.000000
FGF-7	63.64 ±0.4^a^	31.97 ±2.8^b^	32.75 ±5.7^b^	0.000001
HGF	157.09 ±1.6^a^	150.30 ±0.6^b^	45.60 ±2.8^c^	0.001910
TIMP-1	1898.01 ±39.8^a^	1371.93 ±61.2^b^	711.88 ±37.9^c^	0.000000

**Notes.**

H_2_O_2_ alone–cells not incubate with extract; control cell treated only with PBS (no extract, no H_2_O_2_) *N* = 4; *p*-value analysis of variance (ANOVA). Mean ± SD followed by different letters (a,b,c) in the same verse were significantly different for *p* < 0, 05 (post hoc test).

## Discussion

This study showed that methanol-acetone extract from *E. umbellata* fruits protects fibroblasts against the effects of oxidative stress and inflammation triggered by the addition of H_2_O_2_.

### Extract characteristics

HPLC determined that the extract’s main fat-soluble compound was *α*-tocopherol. This finding was in line with previous studies on the content of *E. umbellata* fruit extract. Ahmad, Sabir & Zubair (2006) found that autumn olive fruit possessed 0.9 mg/g d.m. of *α*-tocopherol. The extract in this study contained more than 10 times the concentration of this compound, possibly due to the fact that the extract is much more concentrated than the fruit by itself.

Similarly, the fruit had contained 27.8 mg/100 g d.m. of L-ascorbic acid, while the extract contained 65 mg/100 g d.m., which was in accordance with Khattak’s (2012) findings.

On the other hand, the relatively small amounts of lycopene and beta-carotene probably resulted from the choice of solvent and temperature used during extraction. Since the degree of lycopene extraction increases with temperature (50–60 °C is optimal), acetone, ethyl acetate, or boiling ethanol are the recommended solvents. However, this study’s temperature of extraction was 40 °C, and we used methanol and acetone as solvents in a 80:20 (vol/vol) ratio. Concurrently, beta-carotene has been found to more effectively dissolve in ethanol at lower temperatures (maximum extraction at 25 °C) (Calvo, Dado & Santa-María, 2007). Therefore, extraction conditions were selected to that could also accommodate temperature-sensitive phenols. Khattak (2012) found that *E. umbellata* fruit contained 23.3 mg/g of total phenols, a higher amount than found in our extract. However, their results were given as a gallic acid equivalent, while in our study, we used quercetin as a standard, so the results cannot be easily compared.

#### The extract’s antioxidant potential

The results of our study confirmed that *E. umbellata* fruit extract contained strong anti-oxidative properties. Ozen et al. (2017) found that 500 µg/mL of *E. umbellata* methanol, ethanol, and water extracts had more than 99.5% DPPH scavenging activity, whereas the extract described in this study had 65.55% DPPH scavenging potential. This difference could have been influenced by the use of acetone as a co-solvent for the extract, but also from subtle changes during the experiments (e.g., slight variations in incubation time or laboratory temperature).

The results showed that the extract was a weaker antioxidant than L-ascorbic acid and *α*-tocopherol, which are among the strongest known antioxidants. Although the extract contained both of these vitamins, it should be noted that our extraction protocol did not remove all of the fruit’s non-antioxidant compounds, as the procurement of pure bioactive compounds was outside the scope of the study.

Nutraceuticals and functional foods are becoming increasingly popular and contribute to the public’s growing fear of potential side effects of synthetic compounds. Consumers also have low confidence in supplements containing pure, isolated substances. Therefore, this study focused on evaluating the health benefits of an extract containing a mixture of bioactive compounds and other residues.

#### Influence of the extract and H_2_O_2_ on ROS production in HFFF-2 cells

In this study, we found that *E. umbellata* extract significantly reduced the H_2_O_2_-induced secretion of ROS in HFFF-2 fibroblast cells. As expected, administering H_2_O_2_ significantly increased ROS secretion in the cells. However, pre-treating the cells with the extract caused up to 4 times lower ROS secretion when compared to cells only treated with H_2_O_2_.

The *E. umbellata* extract’s ability to protect cells against oxidative stress might be a result of the presence of phenols, particularly polyphenols, which have the ability to reduce the oxidation rates of organic matter by transferring an H atom (from their OH groups) to chain-carrying ROO* radicals.

Antioxidants can also break radical chains, thereby preventing their propagation. One classic example of a chain-breaking antioxidant is *α*-tocopherol. When delivered to a cell, *α*-tocopherol has been shown to protect membranes against lipid peroxidation (Zhang et al., 2001). Concomitantly, phenols and other compounds that are present in the extract, such as vitamin A and carotenoids, have been reported to scavenge oxygen (Foti, 2007; Moise, Al-Babili & Wurtzel, 2014).

The second possible mechanism is related to antioxidant enzymes. SOD, which scavenges superoxide radicals to H_2_O_2_, is an important endogenous antioxidant enzyme that acts as the first line of defense against ROS, which are further processed by subsequent antioxidant enzymes. Jia et al. (2014) found that SOD activity was reduced in cells after exposure to 500 µM of H_2_O_2_ when compared to the control. This suggests that adding H_2_O_2_ caused oxidative damage to antioxidant enzymes. However, blackcurrant extract limited the decrease in SOD activity of the tested cells (Jia et al., 2014). In this study, 100 µM H_2_O_2_ were applied, which resulted in significantly lower cell mortality than that observed in the Jie et al. study. Accordingly, we showed that a 3 d incubation with extract of *E. umbellata* before the induction of oxidative stress resulted in reduced ROS secretion by HFFF-2 cells. A similar reduction was shown in the SOD activity in these cells, probably owing to the reduced association with oxygen radicals, which are substrates in a SOD catalysed reaction. The *E. umbellata* extract had such strong effects that a reduction in SOD activity was even observed relative to the non-stress induced control.

Administering H_2_O_2_ to the culture medium exposed cells to direct hydroxyl radical (OH) reactions resulting from H_2_O_2_ reacting with iron (II) ions (the Fenton reaction) or with iron and copper ions (the Haber-Weiss reaction). The strong OH oxidizing effect was directed against mitochondrial DNA. The reaction of these metal ions with AA is also important for this process, as L-ascorbic acid acts as a co-substrate of hydroxylases and oxygenases, which are enzymes involved in collagen biosynthesis. Overall, AA keeps the metal ions in a reduced state in the enzyme active site, which in turn enables optimal enzyme activity. L-ascorbic acid reduction of free transition metal ions generates oxygen radicals. Reduced metal ions, such as iron, react with H_2_O_2_to generate highly reactive OH or O_2_^−^ ions. However, due to the limited availability of iron and copper ions in living organism tissue, it has not been confirmed that this reaction takes place in vivo. These metal ions are bound by proteins that are present in cells, such as ferritin, transferrin, and ceruloplasmin (Konopacka, 2004).

The *E. umbellata* extract provides some amounts of calcium and copper ions, but it also contains various antioxidants that may limit L-ascorbic acid’s pro-oxidative effects. Additionally, phenols that are present in the extract have the ability to form stable complexes with transition metal ions, such as Cu^2+^ or Fe^2+^, thus preventing Haber-Weiss and Fenton reactions (Foti, 2007).

#### Influence of the extract and H_2_O_2_ on HFFF-2 cell viability

Additionally, the extract’s effect on the viability of HFFF-2 cells was investigated. Our results showed a significant increase in HFFF-2 cell viability after incubation with *E. umbellata* extract at both tested concentrations, indicating the extract’s safety. This enhanced cell viability was probably related to increased FGF-7 expression. Moreover, the increased HGF expression in cells exposed to the extract further suggested that the extract can affect cell proliferation and differentiation. The *E. umbellata* extract limited cell viability due to the addition of H_2_O_2_, suggesting that it could protect fibroblast cells and even increase proliferation after oxidative stress-induced damage. However, more accurate studies are needed to confirm this result.

#### *E. umbellata* extract rescues fibroblasts from H_2_O_2_-induced cell death

Apoptosis is a non-inflammatory process resulting from the rapid identification and removal of dying cells before they secrete damage-associated molecular patterns (DAMP). In some instances, physiological inducers of apoptosis (i.e., TNF) can promote inflammatory cytokine expression in conjunction with apoptosis, resulting in an apoptotic process that appears pro-inflammatory. Conversely, necrosis is considered pro-inflammatory due to DAMP (Davidovich, Kearney & Martin, 2014).

We evaluated the cell death pathways that were activated by the extract. The percentage of necrosis decreased gradually across all groups except for the non-treated control group. Necrosis was most pronounced in cells treated with only the stress factor (H_2_O_2_), while cells exposed to both the extract and H_2_O_2_ showed significantly lower necrosis rates just 6 h after H_2_O_2_-induced stress. However, only 24 h after H_2_O_2_, administration, the group that received the extract and H_2_O_2_ showed similar necrotic activation as the group that received the extract without the stress factor. These results indicated that H_2_O_2_ causes rapid inflammation-related cellular changes, which HFFF-2 cells can partially neutralize. On the other hand, the extract reduced overall cell death and the incidence of necrosis at rates that were similar to the untreated control group, where no oxidative stress was induced.

#### The *E. umbellata* extract changes the expression of selected growth factors in H_2_O_2_-induced HFFF-2 cells.

During the same oxidative stress testing, we examined the expression of selected cytokines that were associated with inflammation. When compared to cells that were exposed to oxidative stress and did not receive the extract, the use of *E. umbellata* extract decreased expression of the following inflammatory mediators: MIF, fractalkine, MCP-4, BLC, GCP-2, NAP-2, and Eotaxin-2 and 3. As expected, chemokine expression was clearly lower in the control group, which was not treated with H_2_O_2_. Fibroblasts are known to produce inflammatory mediators in response to skin inflammation, similarly to pro-inflammatory cytokines during the progression phase of inflammation.

*E. angustifolia* has been shown to have anti-inflammatory properties when tested on animals. Total and endocarp extracts from this plant decreased formalin–induced paw oedema, similarly to sodium salicylate. Other researchers concluded that analgesia induced by *E. angustifolia* fruit extract did not depend on the opioid system, but flavonoids and terpenoids were the main candidates for the observed analgesic and anti-inflammatory effects (Ahmadiani et al., 2000). Another study showed a decrease in formalin-induced inflammation in mice following treatment with *E. angustifolia* aqueous extract. Moreover, this extract was found to inhibit type 1 and type 2 cyclooxygenase enzymes (Farahbakhsh, Arbabian & Emami, 2011). Liao et al. (2012) found that *Elaeagnus oldhamii maxim* methanol extracts increased SOD and glutathione peroxidase (GPx) activity in liver tissue and decreased malondialdehyde (MDA), nitric oxide (NO), interleukin (IL) 1 β, 6 (IL-6), TNF-*α*, and cyclooxygenase-2 (COX-2) levels in paw oedema tissue after λ-carrageenan-induced inflammatory reactions. Nikniaz et al. (2014) observed the anti-inflammatory properties of *E. angustifolia* fruit powder in women with knee osteoarthritis. They observed a significant decrease in serum pro-inflammatory cytokine (TNF- *α*, MMP-1) levels and a rise in serum anti-inflammatory cytokine (IL-10) levels after 8 weeks of supplementation. No significant changes were observed in the IL-1 β and MMP-13 serum levels (Nikniaz et al., 2014). These in vivo results were consistent with our in vitro results regarding *E. umbellata* extract, although in this study, we studied a larger and more diverse number of cytokines.

The mechanisms that affect pro-inflammatory cytokine expression *in vitro*. Maas, Deters & Hensel (2011) measured the anti-inflammatory activities of *Eupatorium perfoliatum L*. extracts by significantly downregulating the expression of cytokines, including colony stimulating factor 3 (CSF-3), IL-1 *α*, IL-1 β, and the majority of chemokines, such as C-C motif chemokine ligand 2 (CCL2), CCL22 (MCP-1) and C-X-C motif chemokine ligand 10 (CXCL10, IP-10) in LPS-stimulated murine macrophage cells.

Chemokines play an important role in connective tissue diseases by mediating the recruitment of eosinophils to the inflammation site and ROS release, leading to tissue damage and inflammatory response propagation (Elsner et al., 1997). One therapeutic strategy used to reduce excessive inflammatory responses is to inhibit chemokine activity or block their receptors (Waśniowska, 2004). In this study, we observed a decrease in chemokines that were eosinophil chemo-attractants (eotaxins and MCP-4) in oxidative stress-subjected fibroblasts treated with *E. umbellata* extract. Elsner et al. (1997) proposed that using a therapeutical approach that blocks these cytokines would prevent the invasion and destruction caused by eosinophils in certain conditions, such as allergic asthma and connective tissue diseases.

In this study, *E. umbellata* extract decreased the expression of four cytokines that induce MMP expression: NAP-2, CPG-2, MIF/CXCR2, and Fractalkine/CX3CL1. As previously mentioned, MMPs are associated with collagen degradation during oxidative stress. Lee, Seo & Lee (2019) found that Skullcapflavone II (a flavonoid derived from the *Scutellaria baicalensis* root) decreased MMP-1 expression and improved the integrity of type I collagen in foreskin fibroblasts. In recent years, there has been a greater use of natural substances which, instead of reducing MMP expression, increase the expression of their inhibitors (tissue inhibitors of metalloproteinases, TIMPs). The imbalance between MMPs and TIMPs has been characteristic for inflammatory diseases, cancer, and neurodegenerative diseases (Rosenberg, 2009; Calabriso et al., 2016). In this study, *E. umbellata* extract increased TIMP-1 expression by 43% compared to the non-treated control. A similar result was obtained when *Spatholobus suberectus* stem extract was applied to UVB-induced human keratinocyte cells (Kwon et al., 2019), polyphenol-rich red grape skin extract on vascular inflammation cell models (Calabriso et al., 2016), and kuding tea polyphenols on UVB-induced skin aging in hairless mice (Yi et al., 2019).

Proteins from the MPP family are also known to contribute to neurodegenerative diseases by destroying white matter and neurons (Rosenberg, 2009). In addition to increasing MPP inhibitor secretion, *E. umbellata* extract also increased BDNF expression in fibroblast cells under oxidative stress. BDNF is a neurotrophic factor known to prevent axonal and neuronal damage (Stadelmann et al., 2002) and promote the formation of new neurons. HGF has shown a similar effect on cell proliferation in the brain, and also has been reported to regulate the migration and mobility of newly-created cells (Respondek & Buszman, 2015). Insignificant increase in HGF and a marked increase in FGF-7 expression were also observed in our study, suggesting that *E. umbellata* extracts could potentially be used in the treatment of disorders with neuro-inflammation, such as Alzheimer’s or Parkinson’s diseases. However, further research is required on a different model to fully evaluate this.

Additionally, we observed more than a 2-fold increase in FGF-7 expression in cells treated with the extract, which is in accord with the cell viability results that showed an increase in cell proliferation following extract treatment.

Interestingly, TIMP-1, HGF, and FGF-7 expression was lower in the non-treated control group, and BDNF was undetectable. This clearly indicates that *E. umbellata* extract influences the expression of these signalling factors, regardless of oxidative stress.

## Conclusion

This study was the first to explore the antioxidant effects of *E. umbellata* fruit methanol-acetone extract on normal human fibroblasts subjected to oxidative stress. We found that the extracts did not show any toxicity toward HFFF-2 cells at the concentrations used, but instead increased the viability of cells. Additionally, the studied extract reduced cell mortality caused by H_2_O_2_ treatment. We also showed that the extract reduced chemokine expression in H_2_O_2_-induced fibroblast cells. Collectively, our obtained data suggested that *E. umbellata* fruit extract has anti-inflammatory properties and could be used as a nutraceutical (or functional additive) because of its inflammatory-regulating properties. Moreover, the extract was demonstrated to increase the expression of TIMP-1, an MPP-9 inhibitor, indicating that collagen may be protected from degradation during oxidative stress. These properties, combined with the increase in BDNF expression, suggest that *E. umbellata* extracts may be effective in the treatment of neuro-inflammatory disorders, such as Alzheimer’s or Parkinson’s diseases. However, further studies are necessary to delineate the precise mechanisms underlying these activities.

##  Supplemental Information

10.7717/peerj.10760/supp-1 Supplemental Information 1 Raw data

## Additional Information and Declarations

### Data Availability

The following information was supplied regarding data availability: The raw measurements are available in the Supplemental Files

## References

[ref-1] Ahmad H, Khan SM, Ghafoor S, Ali N (2009). Ethnobotanical study of Upper Siran. Journal of Herbs, Spices & Medicinal Plants.

[ref-2] Ahmad SD, Sabir SM, Zubair M (2006). Ecotypes diversity in autumn olive (*Elaeagnus umbellata* Thunb): a single plant with multiple micronutrient genes. Journal of Chemical Ecology.

[ref-3] Ahmadiani A, Hosseiny J, Semnanian S, Javan M, Saeedi F, Kamalinejad M, Saremi S (2000). Antinociceptive and anti-inflammatory effects of Elaeagnus angustifolia fruit extract. Journal of Ethnopharmacology.

[ref-4] Calabriso N, Massaro M, Scoditti E, Pellegrino M, Ingrosso I, Giovinazzo G, Carluccio MA (2016). Red grape skin polyphenols blunt matrix metalloproteinase-2 and -9 activity and expression in cell models of vascular inflammation: protective role in degenerative and inflammatory diseases. Molecules.

[ref-5] Calvo MM, Dado D, Santa-María G (2007). Influence of extraction with ethanol or ethyl acetate on the yield of lycopene, β-carotene, phytoene and phytofluene from tomato peel powder. European Food Research and Technology.

[ref-6] Cusimano A, Balasus D, Azzolina A, Augello G, Emma MR, Sano CDi, Gramignoli R, Strom SC, McCubrey JA, Montalto G, Cervello M (2017). Oleocanthal exerts antitumor effects on human liver and colon cancer cells through ROS generation. International Journal of Oncology.

[ref-7] Davidovich P, Kearney CJ, Martin SJ (2014). Inflammatory outcomes of apoptosis, necrosis and necroptosis. Biological Chemistry.

[ref-8] Elsner J, Petering H, Höchstetter R, Kimmig D, Wells TN, Kapp A, Proudfoot AE (1997). The CC chemokine antagonist Met-RANTES inhibits eosinophil effector functions through the chemokine receptors CCR1 and CCR3. European Journal of Immunology.

[ref-9] Farahbakhsh S, Arbabian S, Emami F (2011). Inhibition of cyclooxygenase type 1 and 2 enzyme by aqueous extract of elaeagnus angustifolia in mice. Autonomic Neuroscience: Basic and Clinical.

[ref-10] Fordham IM, Clevidence BA, Wiley ER, Zimmerman RH (2001). Fruit of autumn olive: a rich source of lycopene. HortScience.

[ref-11] Foti MC (2007). Antioxidant properties of phenols. Journal of Pharmacy and Pharmacology.

[ref-12] Galis ZS, Asanuma K, Godin D, Meng X (1998). N-acetyl-cysteine decreases the matrix-degrading capacity of macrophage-derived foam cells: new target for antioxidant therapy?. Circulation.

[ref-13] Halliwell B, Gutteridge JM (1992). Biologically relevant metal ion-dependent hydroxyl radical generation. An update. FEBS Letters.

[ref-14] Hamidpour R, Hamidpour S, Hamidpour M, Shahlari M, Sohraby M, Shahlari N, Hamidpour R (2016). Russian olive (Elaeagnus angustifolia L.): from a variety of traditional medicinal applications to its novel roles as active antioxidant, anti-inflammatory, anti-mutagenic and analgesic agent. Journal of Traditional and Complementary Medicine.

[ref-15] Jia N, Li T, Diao X, Kong B (2014). Protective effects of black currant (Ribes nigrum L.) extract on hydrogen peroxide-induced damage in lung fibroblast MRC-5 cells in relation to the antioxidant activity. Journal of Functional Foods.

[ref-16] Khattak KF (2012). Free radical scavenging activity, phytochemical composition and nutrient analysis of *Elaeagnus umbellata* berry. Journal of Medicinal Plants Research.

[ref-17] Konopacka M (2004). The role of vitamin C in DNA oxidative damage. Postepy Higieny Medycyny Doswiadczalnej.

[ref-18] Kwon KR, Alam MB, Park JH, Kim TH, Lee SH (2019). Attenuation of UVB-induced photo-aging by polyphenolic-rich spatholobus suberectus stem extract via modulation of MAPK/AP-1/MMPs signaling in human keratinocytes. Nutrients.

[ref-19] Lee YH, Seo EK, Lee ST (2019). Skullcapflavone II inhibits degradation of type i collagen by suppressing MMP-1 transcription in human skin fibroblasts. International Journal of Molecular Sciences.

[ref-20] Liao CR, Chang YS, Peng WH, Lai SC, Ho YL (2012). Analgesic and anti-inflammatory activities of the methanol extract of Elaeagnus oldhamii Maxim, in mice. The American Journal of Chinese Medicine.

[ref-21] Luster AD (1998). Chemokines—chemotactic cytokines that mediate inflammation. The New England Journal of Medicine.

[ref-22] Maas M, Deters AM, Hensel A (2011). Anti-inflammatory activity of Eupatorium perfoliatum L. extracts, eupafolin, and dimeric guaianolide via iNOS inhibitory activity and modulation of inflammation-related cytokines and chemokines. Journal of Ethnopharmacology.

[ref-23] Matusiewicz M, Bączek KB, Kosieradzka I, Niemiec T, Grodzik M, Szczepaniak J, Orlińska S, Węglarz Z (2009). Effect of juice and extracts from saposhnikovia divaricata root on the colon cancer cells Caco-2. International Journal of Molecular Sciences.

[ref-24] Mescher AL (2017). Macrophages and fibroblasts during inflammation and tissue repair in models of organ regeneration. Regeneration.

[ref-25] Moise AR, Al-Babili S, Wurtzel ET (2014). Mechanistic aspects of carotenoid biosynthesis. Chemical Reviews.

[ref-26] Nagata M (2005). Inflammatory cells and oxygen radicals. Current Drug Targets –Inflammation & Allergy.

[ref-27] Nazir N, Zahoor M, Nisar M, Khan I, Karim N, Abdel-Halim H, Ali A (2018). Phytochemical analysis and antidiabetic potential of *Elaeagnus umbellata* (Thunb.) in streptozotocin-induced diabetic rats: pharmacological and computational approach. BMC Complementary and Alternative Medicine.

[ref-28] Nikniaz Z, Ostadrahimi A, Mahdavi R, Ebrahimi AA, Nikniaz L (2014). Effects of Elaeagnus angustifolia L. supplementation on serum levels of inflammatory cytokines and matrix metalloproteinases in females with knee osteoarthritis. Complementary Therapies in Medicine.

[ref-29] Odriozola-Serrano I, Hernández-Jover T, Martín-Belloso O (2007). Comparative evaluation of UV-HPLC methods and reducing agents to determine vitamin C in fruits. Food Chemistry.

[ref-30] Ozen T, Yenigun S, Altun M, Demirtas I (2017). Phytochemical constituents, ChEs and urease inhibitions, antiproliferative and antioxidant properties of elaeagnus umbellata thunb. Combinatorial Chemistry & High Throughput Screening.

[ref-31] Patel S (2015). Plant genus elaeagnus: underutilized lycopene and linoleic acid reserve with permaculture potential. Fruits.

[ref-32] Rao AV, Waseem Z, Agarwal S (1998). Lycopene content of tomatoes and tomato products and their contribution to dietary lycopene. Food Research International.

[ref-33] Respondek M, Buszman E (2015). Regulation of neurogenesis: factors affecting of new neurons formation in adult mammals brain. Postepy Higieny i Medycyny Doswiadczalnej.

[ref-34] Rosenberg GA (2009). Matrix metalloproteinases and their multiple roles in neurodegenerative diseases. The Lancet Neurology.

[ref-35] Schieber M, Chandel NS (2014). ROS function in redox signaling and oxidative stress. Current Biology.

[ref-36] Sies H, Stahl W (1995). Vitamins E and C, beta-carotene, and other carotenoids as antioxidants. The American Journal of Clinical Nutrition.

[ref-37] Song J, Lim H, Kim K, Cho S, Chae S (2012). Effect of caffeic acid phenethyl ester (CAPE) on H2O2 induced oxidative and inflammatory responses in human middle ear epithelial cells. International Journal of Pediatric Otorhinolaryngology.

[ref-38] Spínola V, Pinto J, Llorent-Martínez EJ, Castilho PC (2019). Changes in the phenolic compositions of Elaeagnus umbellata and Sambucus lanceolata after in vitro gastrointestinal digestion and evaluation of their potential anti-diabetic properties. Food Research International.

[ref-39] Stadelmann C, Kerschensteiner M, Misgeld T, Brück W, Hohlfeld R, Lassmann H (2002). Brain.

[ref-40] Szczepaniak J, Strojny B, Sawosz Chwalibog E, Jaworski S, Jagiello J, Winkowska M (2018). Effects of reduced graphene oxides on apoptosis and cell cycle of glioblastoma multiforme. International Journal of Molecular Sciences.

[ref-41] Uddin G, Rauf A (2012). Phytochemical screening and biological activity of the aerial parts of Elaeagnus umbellata. Scientific Research and Essays.

[ref-42] Wang SY, Bowman L, Ding M (2007). Variations in free radical scavenging capacity and antiproliferative activity among different genotypes of autumn olive (Elaeagnus umbellata). Planta Medica.

[ref-43] Waśniowska K (2004). Chemokines –future therapeutic targets. Postępy Higieny i Medycyny Doświadczalnej.

[ref-44] Watanabe H, Shimizu T, Nishihira J, Abe R, Nakayama T, Taniguchi M, Sabe H, Ishibashi T, Shimizu H (2004). Ultraviolet A-induced production of matrix metalloproteinase-1 is mediated by macrophage migration inhibitory factor (MIF) in human dermal fibroblasts. Journal of Biological Chemistry.

[ref-45] Williams IR, Roitt I (1998). Fibroblast. Encyclopedia of immunology.

[ref-46] Yi R, Zhang J, Sun P, Qian Y, Zhao X (2019). Protective effects of kuding tea (Ilex kudingcha C., J. Tseng) polyphenols on UVB-Induced skin aging in skh1 hairless mice. Molecules.

[ref-47] Zhang JG, Nicholls-Grzemski FA, Tirmenstein MA, Fariss MW (2001). Vitamin E succinate protects hepatocytes against the toxic effect of reactive oxygen species generated at mitochondrial complexes I and III by alkylating agents. Chemico-Biological Interactions.

